# Ohio Veterinary College

**Published:** 1896-05

**Authors:** 


### COMMENCEMENT EXERCISES.

### OHIO VETERINARY COLLEGE.

THE Commencement Exercises were held in the college building, Wednesday evening, March 18th. The audience was highly entertained by eloquent speeches by Profs. Frederick Kebler and J. Francis Jones. Prof. George E. Twitchell delivered the valedictory address, and Dr. L. P. Cook presented the diplomas. A banquet concluded the exercises, at which all present averred they enjoyed themselves.

The following are the graduates: George E. Tribou, Owingsville, Ky.; Henry Loth, Cincinnati, O.; D. J. Cargill, Port Jefferson, O.; M. P. Freed, Sharon, Pa.; Butler Smith, Greensburg, Ind.; L. W. Hamilton, Greenvile, Pa.; Roy N. Drake, Oakley, O.

